# The association between micronutrient levels and diabetic foot ulcer: A systematic review with meta-analysis

**DOI:** 10.3389/fendo.2023.1152854

**Published:** 2023-03-29

**Authors:** Shilia Jacob Kurian, Tejaswini Baral, Mazhuvancherry K. Unnikrishnan, Ruby Benson, Murali Munisamy, Kavitha Saravu, Gabriel Sunil Rodrigues, Mahadev Rao, Amit Kumar, Sonal Sekhar Miraj

**Affiliations:** ^1^ Department of Pharmacy Practice, Manipal College of Pharmaceutical Sciences, Manipal Academy of High Education, Manipal, Karnataka, India; ^2^ Manipal Centre for Infectious Diseases, Prasanna School of Public Health, Manipal Academy of Higher Education, Manipal, Karnataka, India; ^3^ NGSM Institute of Pharmaceutical Sciences, NITTE (Deemed to be University), Mangalore, Karnataka, India; ^4^ Department of Translational Medicine, All India Institute of Medical Sciences, Bhopal, Madhya Pradesh, India; ^5^ Department of Infectious Diseases, Kasturba Medical College and Hospital, Manipal, Manipal Academy of Higher Education, Manipal, Karnataka, India; ^6^ Department of General & Laparoscopic Surgery, Aster Al Raffah Hospital, Sohar, Oman; ^7^ Department of Laboratory Medicine, Rajendra Institute of Medical Sciences, Ranchi, Jharkhand, India

**Keywords:** diabetic foot ulcers, micronutrients, vitamins, minerals, risk

## Abstract

**Background:**

Diabetic foot ulcers (DFU) are a major complication of diabetes mellitus (DM). Nutrient deficiencies are among the major risk factors in DFU development and healing. In this context, we aimed to investigate the possible association between micronutrient status and risk of DFU.

**Methods:**

A systematic review (Prospero registration: CRD42021259817) of articles, published in PubMed, Web of Science, Scopus, CINAHL Complete, and Embase, that measured the status of micronutrients in DFU patients was performed.

**Results:**

Thirty-seven studies were considered, of which thirty were included for meta-analysis. These studies reported levels of 11 micronutrients: vitamins B9, B12, C, D, E, calcium, magnesium, iron, selenium, copper, and zinc. DFU, compared to healthy controls (HC) had significantly lower vitamin D (MD: -10.82 14 ng/ml, 95% CI: -20.47, -1.16), magnesium (MD: -0.45 mg/dL, 95% CI: -0.78, -0.12) and selenium (MD: -0.33 µmol/L, 95% CI: -0.34, -0.32) levels. DFU, compared to DM patients without DFU, had significantly lower vitamin D (MD: -5.41 ng/ml, 95% CI: -8.06, -2.76), and magnesium (MD: -0.20 mg/dL, 95% CI: -0.25, -0.15) levels. The overall analysis showed lower levels of vitamin D [15.55ng/ml (95% CI:13.44, 17.65)], vitamin C [4.99µmol/L (95% CI:3.16, 6.83)], magnesium [1.53mg/dL (95% CI:1.28, 1.78)] and selenium [0.54µmol/L (95% CI:0.45, 0.64)].

**Conclusion:**

This review provides evidence that micronutrient levels significantly differ in DFU patients, suggesting an association between micronutrient status and risk of DFU. Therefore, routine monitoring and supplementations are warranted in DFU patients. We suggest that personalized nutrition therapy may be considered in the DFU management guidelines.

**Systematic review registration:**

https://www.crd.york.ac.uk/PROSPERO/display_record.php?RecordID=259817, identifier CRD42021259817.

## 1 Introduction

Chronic wound infections pose a significant health concern, especially diabetic foot ulcers (DFU) with maximum severity. It is estimated that foot ulcer complications account for 24.4% of healthcare costs among diabetics (1). The rising prevalence of diabetes projects DFU as a growing health concern that accounts for maximum non-traumatic amputation globally. Prevalence of DFU among diabetics has risen from 15 - 25% to 19 - 35% (2). The global prevalence of DFU is 6.3%, higher in males and type 2 diabetes mellitus (DM) than in females and type 1 DM (3). A recent study reported the one-, two -, and five-year survival rates in DFU patients as 81%, 69%, and 29%, indicating the robust association with mortality (4). Foot ulcers are less likely to heal in diabetics because of disorders in the intrinsic wound-healing process, such as compromised collagen cross-linking, altered functioning of matrix metalloproteinases, and immunological reasons (5). Management strategies include patient education, wound dressings, debridement, adequate offloading, blood sugar control, infection management, revascularisation, and advanced therapies (6, 7).

Nutrient deficiencies are among the major risk factors in DFU development and healing. Nutrient deficiencies modify the physiological responses to infection by diminishing the immune response, predisposing the skin to become thin and flaky, thereby developing a wound. The deficiencies also decrease subcutaneous fat at pressure points, together exacerbating the vulnerability to pressure wounds. Nutrient deficiencies also reduce the collagen synthesis required for wound healing and promote immobility due to diminished energy reserves (8). Malnutrition adversely affects the complex wound-healing process.

Hyperglycaemia and glucose-lowering drugs alter nutrient absorption in DM patients, resulting in nutritional deficiencies (9). Oxidative stress from glucose metabolism in DM depletes the natural antioxidant reserves of vitamins A, C, and E (9). Persistent hyperglycaemia and open wounds push the body into a catabolic state. As a result of insulin deprivation, negative nitrogen balance develops from gluconeogenesis from protein breakdown. Altered nutritional status and systemic deficiencies impair fibroblast, protein, and collagen synthesis (5).

Micronutrients affect wound healing comprehensively, *via* antioxidant and anti-inflammatory action, collagen stabilization, cell growth regulation, and differentiation. A closer monitoring of micronutrient status in DFU is warranted, as nutrient status is an easily modifiable factor as compared to non-modifiable factors such as age, DM duration, metabolic factors, and micro-, and macro-vascular disorders. The focus of this study was to systematically review the literature and provide the nature of nutritional deficiencies in DFU patients as compared to DM and non-diabetic healthy controls (HC). This would help identify the primary micronutrient deficiencies in DFU patients and initiate supplementations accordingly. Therefore, we have collated and analysed multiple data related to micronutrient status in patients with DFU, DM, and healthy controls (HC).

## 2 Methods

This systematic review appraises the association between micronutrient status and the risk of DFU. We have followed the preferred reporting items for systematic review and meta-analysis (PRISMA) 2020 guidelines and developed the research question using the PECOS format: The original research articles (study design) among DFU patients (participants), micronutrient status (exposure) as a risk for foot ulcers (outcome) compared to the control groups (comparator). The study protocol was registered in the International Prospective Register of Systematic Reviews (PROSPERO), identification number CRD42021259817 (https://www.crd.york.ac.uk/PROSPERO/display_record.php?RecordID=259817).

### 2.1 Search strategy

Initial search was performed in July 2021 and updated on 21^st^ October 2021. We systematically searched and identified relevant studies from the following databases: PubMed, Web of Science, Scopus, CINAHL Complete, and Embase. The references cited by the included articles were examined to identify more articles. We used the following search terms: ‘micronutrient*’, ‘nutrient*’, ‘nutritional status’, ‘trace element*’, ‘vitamin*’, ‘provitamin*’, ‘mineral’, ‘diabetic foot ulcer*’, ‘DFU’, ‘diabetic foot infection*’, ‘diabetic foot osteomyelitis’, ‘diabetic foot’, ‘diabetic feet’ combined using ‘AND’ and ‘OR’, without restrictions on date of publication and language.

### 2.2 Eligibility and study selection

The study titles and abstracts were initially screened, and full texts were examined for potential eligibility. We included studies published in English and all original research studies (RCTs and observational studies) that measured micronutrient status in DFU without date restrictions. Only baseline data regarding the demographics and micronutrient levels in DFU patients were retrieved from RCTs. We excluded animal studies, editorials, case reports, case series, abstract-only papers, conference proceedings, and publications that did not measure micronutrient levels. After the initial search, all references were downloaded to Endnote X9.3.3 software. Further, SJK and RB independently assessed the title and abstracts to check for eligibility based on inclusion and exclusion criteria. Disagreements were resolved by SSM.

### 2.3 Data extraction and quality assessment

Data from the included studies were extracted into a pre-framed data extraction sheet. The following variables were extracted: author name(s), year of publication, place of study, study design, patient demographic characteristics, number of patients in cases/control, sample size, DFU classification, and micronutrient assessed and status of micronutrient. SJK performed primary data extraction, which was cross-checked for accuracy by TB and RB. Disagreements were resolved by discussion/consultation with SSM. For RCTs, only the baseline micronutrient levels were extracted.

We used Cochrane risk-of-bias tool to assess the quality of RCTs, the Newcastle-Ottawa Scale (NOS) for observational studies (e.g., case-control and cohort studies), and Joanna Briggs Institute (JBI) critical appraisal checklist for cross-sectional studies. SJK and TB independently performed the quality assessment, and disagreements between reviewers were settled through consensus/discussion with SSM.

### 2.4 Statistical analysis

From extracted data, we developed a narrative synthesis structured around micronutrient status, findings are presented in tabular form. We employed RevMan 5.4.1 software to perform meta-analysis of selected studies with quantitative estimation.

All data were systematically collected and converted to standard units to maintain uniformity of data using conversion tools (10). We used the statistics toolkit (STATTOOLS) developed by The Department of Obstetrics and Gynaecology of the Chinese University of Hong Kong (11) to combine the mean and standard deviation (SD), where cases or controls were categorized into multiple groups. The formula $SE=SD÷\sqrt{sample\;size}$ was used to convert SD to standard error (SE) and vice versa as per Cochrane guideline (12).

Studies reporting vitamin E were excluded from the meta-analysis because we could not convert multiple units of measurement into a standardized uniform unit. Similarly, zinc values from Momen-Heravi et al. study were excluded from the meta-analysis (13). Unit mismatches could be due to the differences in analytical methods. We excluded vitamin D levels reported by Qasim et al. from the review because it had the lowest score in quality assessment (14). We also excluded vitamin D levels reported by Greenhagen et al. from meta-analysis because SD values were not mentioned (15).

The I^2^ statistic was used to identify the heterogeneity among studies. A random-effects meta-analyses model was conducted because there was significant heterogeneity (I^2^>50%; *P*<0.01) in all the analyses performed. Subgroup analysis was carried out based on the geographical location, but not age and gender because of insufficient data.

### 2.5 Publication bias and sensitivity analysis

The publication bias was assessed using funnel plots. Based on the risk assessment scores, sensitivity analysis was performed to ensure the robustness of the data.

## 3 Results

**Figure 1:**
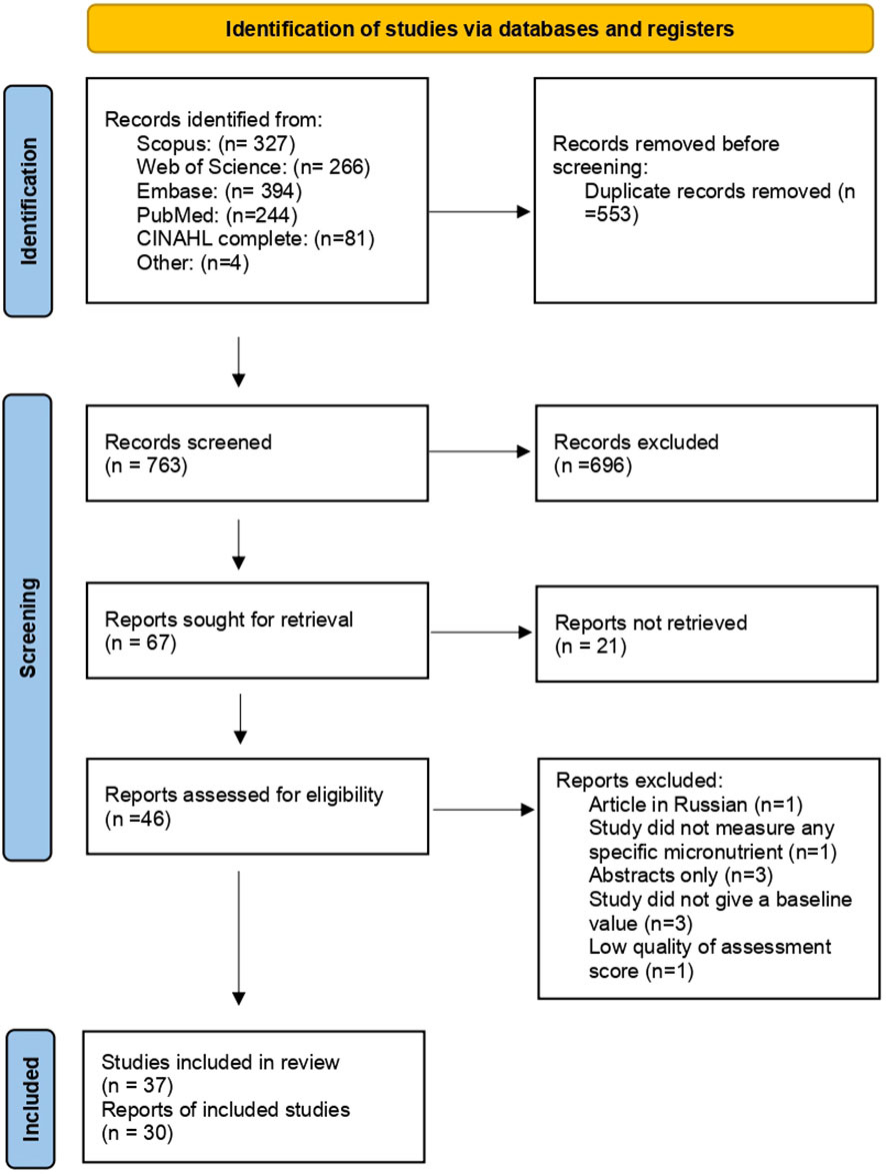
The PRISMA flow chart of screening and study selection.

We identified 1312 records from the databases listed. We identified four more relevant studies by manually searching literature references. We removed 553 duplicate records. The remaining 763 were screened based on title and abstract, of which 67 were selected for retrieval. Finally, a total of 46 articles were assessed for eligibility based on criteria, of which 9 were excluded as some were abstract only (n=3), baseline micronutrient levels were not reported (n=3), a specific micronutrient assessment was not made (n=1), low-quality assessment score (n=1), and an article was not in English. 37 were included in the review and 30 for meta-analysis. **Figure 1**: The PRISMA flow chart of study selection.

### 3.1 Study characteristics

A total of 37 articles were retrieved after a systematic literature search. Nine were RCTs (13, 16–23), and 28 were observational studies (15, 24–50) (12 cross-sectional, seven cohort, and nine case-control studies).

Nine (24.32%) each were reported from India (18, 25, 26, 30, 40, 47–50), and Iran (13, 16, 20–22, 24, 34, 39, 45), three (8.10%) from Turkey (27, 36, 43), two (5.40%) each from China (33, 37) and Nigeria (41, 42), and one (2.70%) each from Italy (19), Bulgaria (31), Greece (32), Pakistan (28), Bahrain (29), USA (15), Germany (38), Australia (35), Spain (44), Mexico (46), Denmark (17), and Slovakia (23). Number of DFU patients (men and women) ranged from 19 to 387. Multiple classification systems were used for DFU assessment such as University of Texas Wound Classification System, Wagner’s grading system, International Working Group on the Diabetic Foot (IWGDF) guideline 2019, and Armstrong classification of chronic wounds, and some were based on the clinical characteristics of the wound. These studies reported levels of 11 nutrients: vitamins B9, B12, C, D, E, calcium, magnesium, iron, selenium, copper, and zinc. **Table 1** provides the study characteristics.

**Table 1:** Study Characteristics.

Sl. no	Year, study title, place	Participants included	Micronutrients assessed	DFU assessment	Major findings
1.	2017 Momen-Heravi et al., Iran (12)	DFU- 60	Zinc	Wagner’s grading system	Zinc supplementation significantly improved wound status and various biochemical markers.
2.	2019 Greenhagen et al., USA (14)	DFU-54 DM- 46	Vitamin D	NA	Significant VDD was identified in patients with various lower extremity complications, with and without ulcers.
3.	2019 Afzali et al., Iran (15)	DFU- 57	Magnesium	Wagner’s grading system	Evident decrease in magnesium levels in DFU. Magnesium and vitamin E supplementation significantly improved wound healing and biochemical markers.
4.	2021 Halschou-Jensen et al., Denmark (16)	DFU- 48	Vitamin D	Based on clinical characteristics of the wound	VDD was markedly prevalent in DFU. High-dose vitamin D (6800IU/day) with standard care achieved a 100% median wound reduction.
5.	2020 Kamble et al., India (17)	DFU- 60	Vitamin D	Wagner’s grading system	VDD was markedly prevalent in DFU. Vitamin D supplementation provided positive outcomes in wound healing and biochemical markers.
6.	2014 Maggi et al., Italy (18)	DFU- 30	Vitamin D	NA	VDD was markedly prevalent in the study population.
7.	2018 Razzaghi et al., Iran (19)	DFU- 70	Magnesium	Wagner’s grading system	Magnesium supplementation significantly improved wound status and various biochemical markers.
8.	2017 Razzaghi et al., Iran (20)	DFU- 60	Vitamin D	Wagner’s grading system	VDD was markedly prevalent in the study population. Positive outcomes in wound healing and biochemical markers upon vitamin D supplementation.
9.	2016 Mozaffari-Khosravi et al., Iran (21)	DFU- 27	Vitamin D	Wagner’s grading system	Both 150,000 and 300,000 IU of vitamin D improved ulcer characteristics, inflammatory, glycemic, and vitamin D status in DFU. 300,000 IU was found more effective than 150,000IU.
10.	2010 Palacka et al., Slovakia (22)	DFU- 59	Vitamin E	Wagner’s grading system	Administration of polarised light along with antioxidant nutrients enhances outcomes in diabetic complications.
11.	2016 Afarideh et al., Iran (23)	DFU- 30 DM- 30 HC- 28	Vitamin D	University of Texas Wound Classification System	Serum 25(OH)D was higher in DFU than in DM and HC. Positive correlation between higher vitamin D levels and the risk of DFU.
12.	2019 Darlington et al., India (24)	DFU- 88 DM- 88	Vitamin D	Wagner’s grading system	Vitamin D was less than 30ng/ml in 59.18% with a graft or achieved wound healing and in 97.44% of patients who either died or needed an amputation. 78.9% with healed wounds within six months had normal levels.
13.	2016 Gupta et al., India (25)	DFU- 50 DM- 50 HC- 25	Vitamin D	NA	Serum vitamin D levels were significantly lower in DFU than in controls. Vitamin D augments phagocytosis by macrophages and thereby enhances the innate immune response.
14.	2013 Keskek et al., Turkey (26)	DFU- 49 DM- 49 HC- 49	Magnesium	Based on clinical characteristics of the wound	A robust association between serum magnesium and incidence of DFU. Significantly lower magnesium in DFU compared to DM and HC.
15.	2020 Shaikh et al., Pakistan (27)	DFU- 387	Calcium	Wagner’s grading system	Mini-nutritional assessment scores were correlated to DFU severity. No correlation between calcium levels and foot ulcers.
16.	2019 Smart et al., Bahrain (28)	DFU- 80	Vitamin D	Wagner’s grading system	85% of study participants had <20ng/ml vitamin D. VDD to be included among the modifiable DFU aggravating factors.
17.	2012 Swain et al., India (29)	DFU- 74	Vitamin D Calcium	NA	Serum vitamin D < 20ng/ml; risk of vascular calcification higher with levels <10ng/ml.
18.	2020 Todorova et al., Bulgaria (30)	DFU- 73 DM- 169	Vitamin D	International Working Group on the Diabetic Foot guideline 2019	VDD significant in DFU. No significant difference in vitamin D levels between infected and uninfected ulcers.
19.	2020 Tsitsou et al., Greece (31)	DFU- 33 DM- 35 HC- 28	Vitamin D Calcium	Based on clinical characteristics of the wound	Significant VDD in diabetic patients with and without ulcers compared to HC
20.	2020 Xiao et al., China (32)	DFU- 245 DM- 4039	Vitamin D	NA	Significant VDD in DFU patients.
21.	2021 Yarahmadi et al., Iran (33)	DFU- 32	Vitamin D	NA	Increased hs-CRP, prooxidant-antioxidant balance, and decreased vitamin D levels could affect the pathogenesis of DFU.
22.	2020 Brookes et al., Australia (34)	DFU- 48	Vitamin D Iron Zinc Selenium Vit C Vitamin B12	NA	More than 50% of participants had VDD and vitamin C deficiency. The risk of amputation is associated with lower levels of vitamin C, albumin, and hemoglobin. The duration of the ulcer is unaffected by nutritional markers.
23.	2018 Caglar et al., Turkey (35)	DFU- 58 DM- 47	Vitamin D	Wagner’s grading system	Vitamin D significantly decreased in DFU; vitamin D supplements might avoid untoward immunological responses.
24.	2020 Dai et al., China (36)	DFU- 21 DM-30	Vitamin D	University of Texas Wound Classification System	VDD is a risk factor for DFU. A cut-off value of 13.68 ng/ml of 25 (OH) vitamin D as the threshold for DFU risk.
25.	2018 Feldkamp et al., Germany (37)	DFU- 104 DM- 103 HC- 99	Vitamin D	Armstrong classification of chronic wounds	Significant VDD in DFU patients; severe VDD in more than half, indicating DFU patients to be at risk for VDD.
26.	2019 Najafpour et al., Iran (38)	DFU- 35 DM- 35 HC- 35	Vitamin D	Wagner’s grading system	Significant VDD in DFU patients. VDD is a risk factor for the development and formation of ulcers in DM.
27.	2013 Zubair et al., India (39)	DFU- 90162 DM- 162	Vitamin D	University of Texas Wound Classification System	Median vitamin D levels are lower in foot ulcer group than in controls. Multivariate analysis showed that low vitamin D predicted foot ulcers.
28.	2016 Bolajoko et al., Nigeria (40)	DFU- 70 HC- 50	Vitamin C Vitamin E Copper Zinc Selenium	Wagner’s grading system	Vitamin C, vitamin E, and selenium are significantly lower in ulcer patients. But copper and zinc levels were similar for all participants.
29.	2012 Bosede et al., Nigeria (41)	DFU- 50 HC- 50	Selenium Vitamin C Vitamin E	Wagner’s grading system	Vitamin C, vitamin E, and selenium lower in DFU than in HC.
30.	2013 Bozkurt et al., Turkey (42)	DFU- 50 DM- 50 HC- 100	Copper Zinc Magnesium	NA	Possible association between elevated zinc levels and DFU. Serum copper and zinc were higher in the DFU and DM than in HC (P<0.001). Serum magnesium was lower in all diabetic patients.
31.	2010 Gonz´alez et al., Spain (43)	DFU- 89 DM- 109	Folate Vitamin B12	Wagner’s grading system	Vitamins folate and B12 levels were similar in both DFU and DM.
32.	2007 Larijani et al., Iran (44)	DFU- 19 DM- 20 HC- 20	Zinc	NA	Serum zinc is significantly lower in DFU; possibly contributing to the hyperactivity of polymorphonuclear leukocytes.
33.	2001 Rodrigues-Moran et al., Mexico (45)	DFU- 33 DM- 66	Magnesium	Based on clinical characteristics of the wound	Significantly lower serum magnesium levels among the DFU.
34.	2013 Tiwari et al., India (46)	DFU- 125 DM- 164	Vitamin D	NA	VDD was substantially more prevalent and severe in DFI than in controls. VDD is a possible risk factor. Initiating supplementation improves patient outcomes.
35.	2014 Tiwari et al., India (47)	DFU- 112 DM- 107	Vitamin D	Wagner’s grading system	Severe VDD in DFI patients is also associated with increased inflammatory cytokines. A cut-off value of 10ng/ml of 25 (OH) vitamin D for immunological alterations in DM patients.
36.	2020 Yadav et al., India (48)	DFU- 32 DM- 32	Zinc, Magnesium Copper	Based on clinical characteristics of the wound	Serum zinc, copper, and magnesium levels were substantially reduced in DFU and also found to be inversely related to glycaemic parameters and directly proportional to the duration of DM.
37.	2008 Singh SK et al., India (49)	DFU- 32 DM- 15 HC- 15	Vitamin E	NA	Diabetic patients with PVD and foot ulcers had significantly lower antioxidant levels and vitamin E.

VDD, vitamin D deficiency; NA, not available; DFU, Diabetic Foot Ulcer; DFI, Diabetic Foot Infections; DM, Diabetes mellitus; HC, Healthy controls; PVD, Peripheral Vascular Disease.

### 3.2 Quality assessment

We employed the Cochrane risk-of-bias tool to assess the quality of RCTs. Case-control and cohort studies were assessed using the NOS. The overall NOS scores for the cohort and case-control studies were 5 to 7, and 6 to 8, respectively, indicating moderate quality. We used JBI checklist for cross-sectional studies. The highest and lowest scores were 8, and 2. Qasim et al. (lowest score) was excluded (14). **Table 2** lists the Quality assessment scores of all included studies.

**Table 2:** Risk of bias assessment using Cochrane risk-of-bias tool for RCTs and Newcastle-Ottawa scale for observational studies, and Joanna Briggs Institute (JBI) critical appraisal checklist for cross-sectional studies.

RCT- Cochrane risk-of-bias																
Study		Sequence generation		Allocation concealment	Blinding of participants and personnel			Blinding of outcome assessment		Incomplete outcome data		Selective reporting			Other bias	
**Momen-Heravi M_2017** (12)		Low		Unclear	Unclear			Unclear		Low		Low			Low	
**Afzali_2019** (15)		Low		High	Low			Low		Low		Low			Low	
**Halshchou-Jensen_2021** (16)		Low		Low	Unclear			Unclear		Low		Low			Low	
**Kamble_2020** (17)		High		High	High			High		Low		Low			Low	
**Maggi_2014** (18)		Low		High	Unclear			Unclear		Unclear		Low			Low	
**Razzaghi_2018** (19)		Low		Low	Unclear			Unclear		Low		Low			Low	
**Razzaghi_2017** (20)		Low		Low	Unclear			Unclear		Low		Low			Low	
**Mozaffari-Khosravi_2016** (21)		Low		High	Unclear			Unclear		Low		Low			Low	
**Palacka_2010** (22)		High		High	High			High		Unclear		Low			Unclear	
Cohort study- Newcastle-Ottawa scale																
Study	Selection								Comparability		Exposure					Final Score
	Representativeness		Selection			Ascertainment	Demonstration		Comparability		Assessment		Duration	Adequacy		
**Greenhagen_ 2019** (14)	0		0			1	1		2		1		0	0		5
**Brookes_ 2020** (34)	1		0			1	0		2		1		0	0		5
**Caglar_ 2018** (35)	1		1			1	1		1		1		0	0		6
**Dai_ 2020** (36)	1		1			1	1		2		1		0	0		7
**Feldkamp_ 2018** (37)	1		1			1	0		1		1		0	0		5
**Najafpour_ 2019** (38)	1		1			1	0		1		1		0	0		5
**Zubair_2013** (39)	1		1			1	1		2		1		0	0		7
Case-control study- Newcastle-Ottawa scale																
Study	Selection								Comparability		Exposure					Final Score
	Case definition		Representativeness			Selection of Controls	Definition of Controls		Comparability		Ascertainment		Method of ascertainment	Non-Response rate		
**Bolajako_ 2016** (40)	1		1			1	1		2		1		0	0		7
**Bosede_ 2012** (41)	1		1			0	1		2		1		1	0		7
**Bozkurt_2013** (42)	0		1			0	1		2		1		1	0		6
**Gonz´alez_2010** (43)	1		1			0	1		2		1		1	1		8
**Larijani_2007** (44)	1		1			1	1		2		1		1	0		8
**Rodrigues-Moran_ 2001** (45)	1		1			1	1		2		1		1	0		8
**Tiwari_ 2013** (46)	1		1			0	1		0		1		1	1		6
**Tiwari_ 2014** (47)	1		1			1	1		0		1		1	0		6
**Yadav_ 2020** (48)	1		1			0	1		1		1		1	1		7
Cross-sectional study- Joanna Briggs Institute critical appraisal checklist																
	Were the criteria for inclusion in the sample clearly defined?		Were the study subjects and the setting described in detail?			Was the exposure measured in a valid and reliable way?	Were objective, standard criteria used for measurement of the condition?		Were confounding factors identified?		Were strategies to deal with confounding factors stated?		Were the outcomes measured in a valid and reliable way?	Was appropriate statistical analysis used?		
**Qasim_ 2020** (13)	No		Yes			Unclear	No		No		No		Yes	Unclear		exclude
**Afarideh_2016** (23)	Yes		Yes			Yes	Yes		Yes		Yes		Yes	Yes		Include
**Darlington_2019** (24)	Yes		Yes			Yes	Yes		No		No		Yes	Yes		Include
**Gupta_ 2016** (25)	Unclear		Yes			Yes	Yes		No		No		Yes	Yes		Include
**Kenskek_2013** (26)	No		Yes			Yes	Yes		No		No		Yes	Yes		Include
**Shaikh_ 2020** (27)	Yes		Yes			Yes	Yes		No		No		No	Yes		Include
**Smart_ 2019** (28)	Unclear		Yes			Yes	Yes		Yes		Yes		Yes	Yes		Include
**Swain_ 2012** (29)	No		No			Yes	Yes		No		No		Yes	Yes		Include
**Todorova_2020** (30)	Yes		Yes			Yes	Yes		No		No		Yes	Yes		Include
**Tsitsou_ 2020** (31)	Yes		Yes			Yes	Yes		No		No		Yes	Yes		Include
**Xiao_ 2020** (32)	Yes		Yes			Yes	Yes		Yes		Yes		Yes	Yes		Include
**Yarahamadi_2021** (33)	No		Unclear			Yes	Yes		No		No		Yes	Yes		Include
**Singh_ 2008** (49)	Unclear		Unclear			Yes	Yes		No		No		Yes	Yes		Include

### 3.3 Meta-analysis

**Figure 2:**
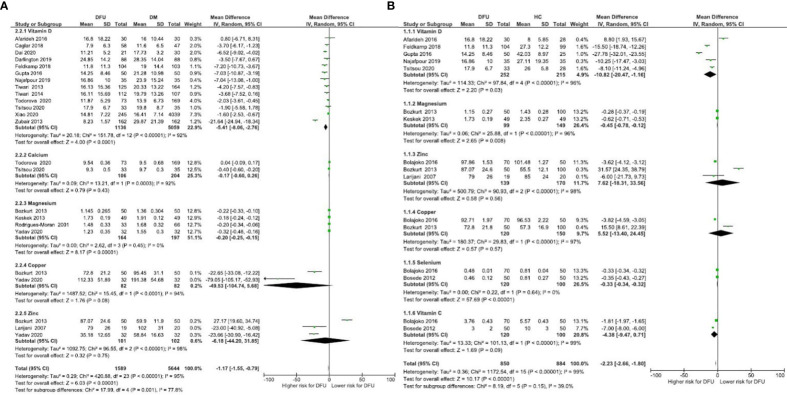
Forest plot of pooled mean difference of micronutrient status in DFU patients compared to DM and HC **(A)** Micronutrient levels of DFU patients were compared against those with DM; **(B)** Micronutrient levels of DFU patients were compared against those with HC.

**Figure 3:**
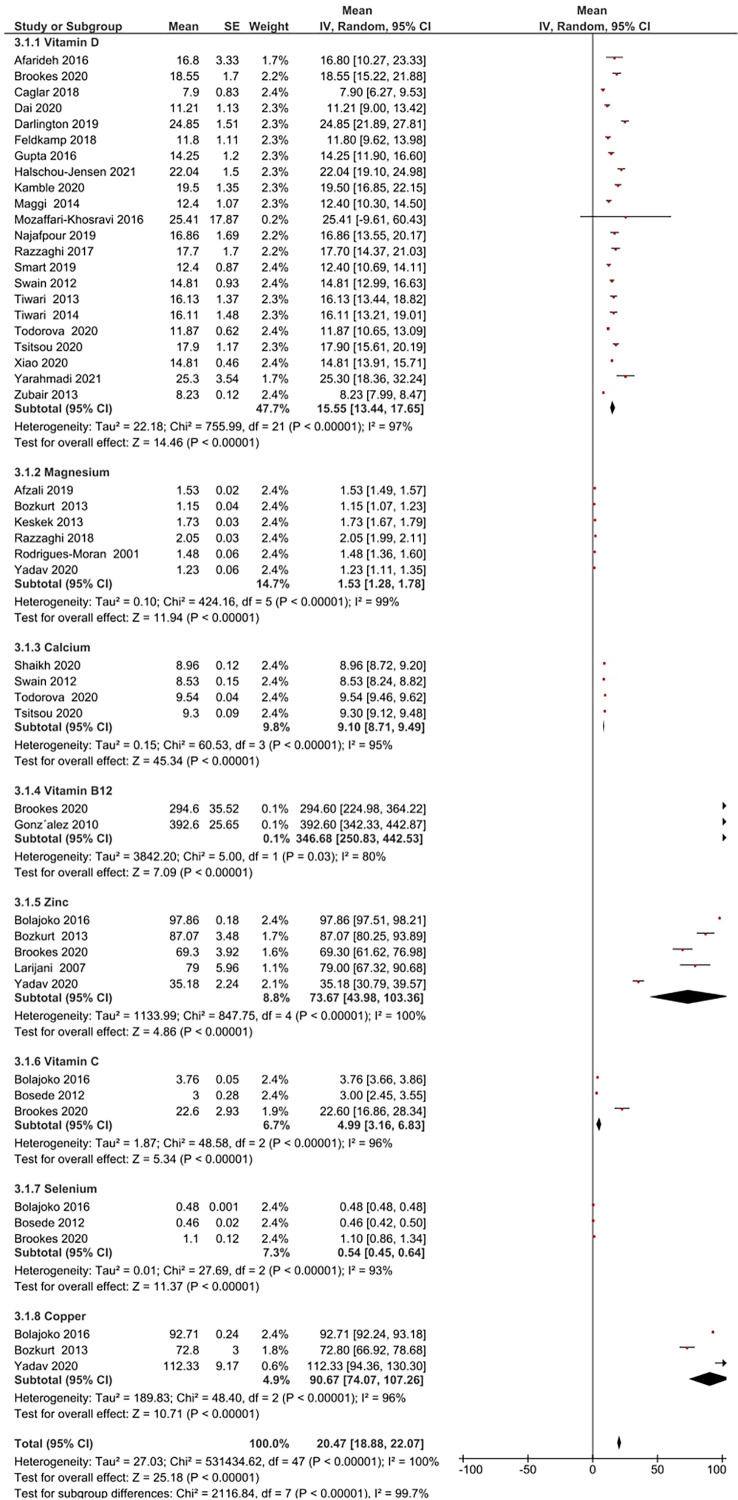
Summary of micronutrient levels in DFU patients. Forest plot of mean micronutrient status in DFU patients.

Micronutrient levels of DFU patients were compared against those with DM [**Figure 2A**] and HC [**Figure 2B**] and are reported in mean differences (MD). **Figure 3** presents the summary results of micronutrient levels in DFU patients.

#### 3.3.1 Vitamin B

Gonzalez et al. estimated folic acid and vitamin B12 levels among DM (n= 109) and DFU (n= 89) patients (44). Serum folic acid (24.9 ± 11.51 vs 25.8 ± 16.6 nmol/L, *P* = 0.67), and vitamin B12 (392.6 ± 242 vs 453.9 ± 290.8 pmol/L, *P* = 0.15) were similar in both groups. Brookes et al. reported vitamin B12 in DFU (n= 39) patients with a mean 294.6 ± 221.8 pmol/L (35). The pooled vitamin B12 level in DFU (n= 128) patients was 346.68 pmol/L, 95% CI: 250.83, 442.53; *P*<00001; *I* ^2^ = 80%.

#### 3.3.2 Vitamin C

Two studies compared vitamin C in DFU (n=120) and HC (n= 100) patients (41, 42). Combined results showed no significant difference in vitamin C levels between the two groups (MD: -4.38 µmol/L, 95% CI: -9.47, 0.71; *P*= 0.09; *I* ^2^ = 99%). A total of three studies measured vitamin C in patients with DFU (35, 41, 42). The mean vitamin C level in DFU (n= 166) patients was 4.99 µmol/L, 95% CI: 3.16, 6.83; *P*<00001; *I* ^2^ = 96%.

#### 3.3.3 Vitamin D

Thirteen studies compared vitamin D levels in DFU (n= 1136) and DM (n= 5059) patients (24–26, 31–33, 36–40, 47, 48). Combined results showed significantly lower vitamin D levels in DFU patients (MD: -5.41 ng/ml, 95% CI: -8.06, -2.76; *P*<0001; *I* ^2^ = 92%). Combined results of five studies in DFU (n= 252) and HC (n= 215) (24, 26, 32, 38, 39); show significantly lower vitamin D levels in DFU (MD: -10.82 14 ng/ml, 95% CI: -20.47, -1.16; *P*=0.03; *I* ^2^ = 96%). From 22 studies that measured vitamin D in patients with DFU (n= 1433) (17–19, 21, 22, 24–26, 29–40, 47, 48), mean levels in patients were 15.55ng/ml, 95% CI: 13.44, 17.65; *P*<00001; *I* ^2^ = 97%. Greenhagen et al. reported 18.7ng/ml of vitamin D in 54 DFU patients compared to 23.6 ng/ml in DM (n= 46) patients without ulcers (15).

#### 3.3.4 Vitamin E

Four studies estimated Vitamin E. Singh et al. measured vitamin E levels in DFU (n= 32) patients, DM (n= 15), and HC (n= 15) (50). Vitamin E levels were substantially lower in DFU, compared to DM (5.04 ± 1.76 vs. 9.10 ± 2.83 ng/L, *P*<0.001) and HC (10.68 ± 2.58ng/L). Bolajoko et al. found lower vitamin E levels in DFU (n= 120) vs DM (n= 50) 19.57 ± 1.01 vs 25.57 ± 0.27 µmol/L, *P*= 0.0001 (41). A study by Bosede et al. demonstrated no significant difference in vitamin E between DFU (n= 50) and HC (n=50) (0.05 ± 0.02 vs. 0.06 ± 0.005 mmol/L) (42). Palacka et al. assessed multiple baseline metabolic parameters in DFU patients, among which vitamin E was 18.48 ± 7.62 mmol/L (23).

#### 3.3.5 Calcium

Two studies compared calcium levels in DFU (n= 106) and DM (n= 204) patients (31, 32). The combined results showed similar calcium levels in both groups (MD: -0.17 mg/dL, 95% CI: -0.60, 0.26; *P*=0.43; *I* ^2^ = 92%). A total of four studies measured calcium in DFU (n=567) (28, 30–32), with mean levels of 9.10 mg/dL, 95% CI: 8.71, 9.49; *P*<00001; *I* ^2^ = 95%.

#### 3.3.6 Magnesium

Combined results from 4 studies comparing magnesium levels in DFU (n= 164) and DM (n= 197) patients (27, 43, 46, 49); showed lower magnesium levels in DFU (MD: -0.20 mg/dL, 95% CI: -0.25, -0.15; *P*<00001; *I* ^2^ = 0%). Combined results of two other comparison studies in DFU (n= 99) and HC (n= 149) patients (27, 43); showed lower magnesium levels in DFU patients (MD: -0.45 mg/dL, 95% CI: -0.78, -0.12; *P*=0.008; *I* ^2^ = 96%). From total of six studies (16, 20, 27, 43, 46, 49], pooled magnesium level was 1.53mg/dL, 95% CI: 1.28, 1.78; *P*<00001; *I* ^2^ = 99% in DFU (n= 291).

#### 3.3.7 Iron

Only one study reported Iron levels. A retrospective analysis by Brookes et al. reported mean iron levels of 8.4 ± 5.9 µmol/L in 29 DFU patients (35).

#### 3.3.8 Selenium

Combined results of two studies comparing selenium in DFU (n=120) and HC (n=100) (41, 42); showed significant difference in selenium levels between both groups (MD: -0.33 µmol/L, 95% CI: -0.34, -0.32; *P*< 0.00001; *I* ^2^ = 0%). A total of three studies measuring selenium in DFU (n=123) (35, 41, 42), reported mean levels of 0.54 µmol/L, 95% CI: 0.45, 0.64; *P*<00001; *I* ^2^ = 93%.

#### 3.3.9 Copper

Combined results of two studies comparing copper levels in DFU (n=82) and DM (n=82) (43, 49) showed similar copper levels in both groups (MD: -49.53 μg/dL, 95% CI: -104.74, 5.68; *P*= 0.08; *I* ^2^ = 94%). Combined results of two studies comparing copper levels in DFU (n= 120) and HC (n= 150) (41, 43); showed similar levels in both groups (MD: 5.52 μg/dL, 95% CI: -13.40, 24.45; *P*=0.57; *I* ^2^ = 97%). Three studies measuring copper in DFU (n= 152) (41, 43, 49), reported mean levels of 90.67 μg/dL, 95% CI: 74.07, 107.26; *P*<00001; *I* ^2^ = 96%.

#### 3.3.10 Zinc

Combined results of three studies comparing zinc levels in DFU (n=101) and DM (n= 102) patients (43, 45, 49) showed similar levels in both groups (MD: -6.18 μg/dL, 95% CI: -44.20, 31.85; *P*=0.75; *I* ^2^ = 98%). Combined results of three studies comparing zinc levels in DFU (n= 139) and HC (n=170) (41, 43, 45); showed similar levels in both groups (MD: 7.62 μg/dL, 95% CI: -18.31, 33.56; *P*= 0.56; *I* ^2^ = 98%). A total of five studies measuring zinc in patients with DFU (n= 180) (35, 41, 43, 45, 49) reported overall level of 73.67 μg/dL, 95% CI: 43.98, 103.36; *P*<00001; *I* ^2^ = 100%. One RCT by Momen-Heravi et al. on the effect of zinc supplements in DFU patients reported the baseline zinc level as 77 ± 9.60 mg/dL (13).

### 3.4 Subgroup analysis, sensitivity analysis, and publication bias

**Figure 4:**
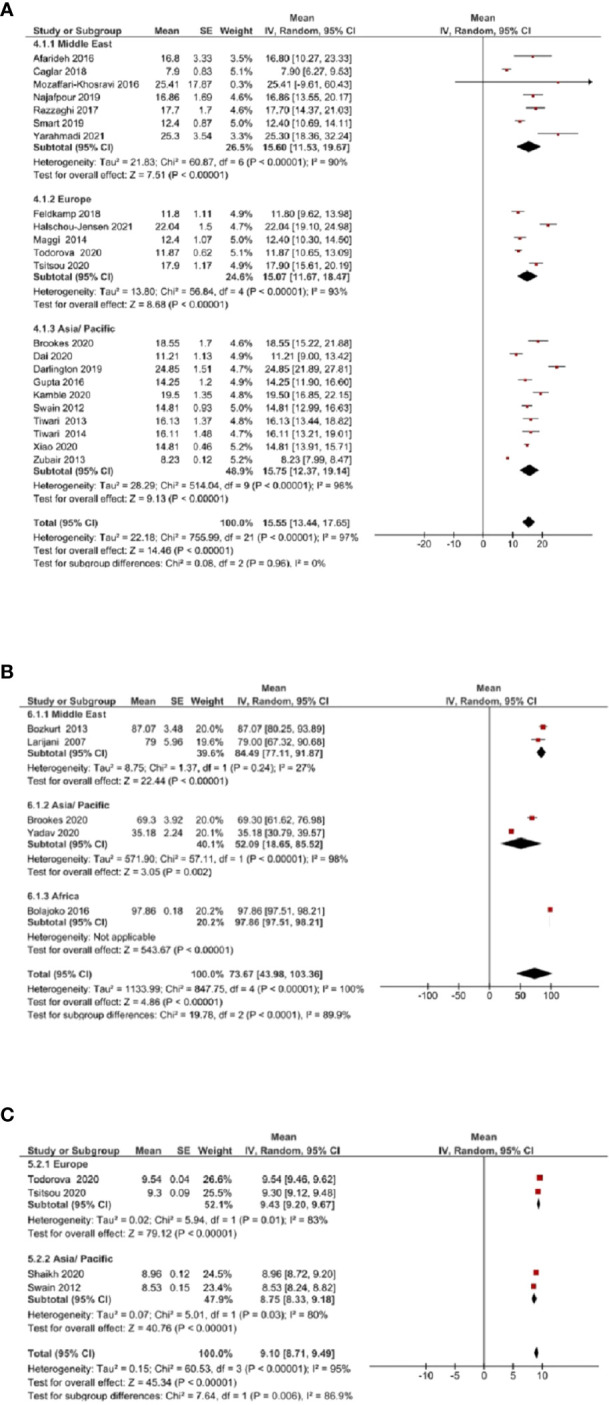
Subgroup analysis based on geographic location was assessed for vitamin D, zinc, and calcium. **(A)** Mean vitamin D levels across Middle East, Europe, and Asia/Pacific regions. **(B)** Mean zinc levels across Middle East, Asia/Pacific, and African regions. **(C)** Mean calcium levels across Europe and Asia/Pacific regions.

Due to insufficient data, subgroup analysis (based on geographic location) was conducted only for vitamin D, zinc, and calcium. The mean vitamin D levels [(**Figure 4A**] were not significantly different across Middle East, Europe, and Asia/Pacific regions (*P*=0.96). Mean zinc levels [(**Figure 4B**] significantly differed between Middle East, Asia/Pacific, and African regions (*P*<0.0001). The mean calcium levels [(**Figure 4C**] differed significantly between Europe and Asia/Pacific regions (*P*=0.006).

**Figure 5:**
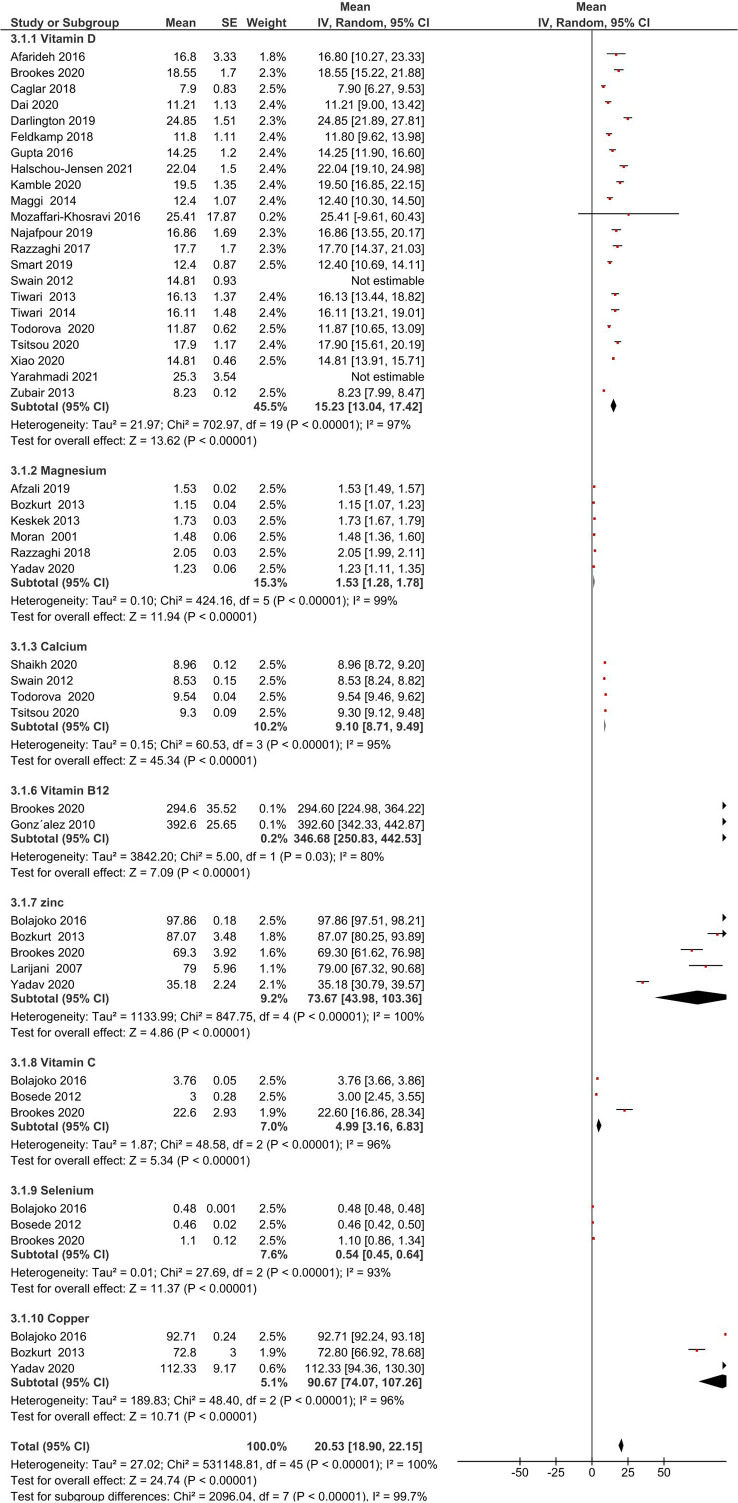
Forest plot for the sensitivity analysis. Sensitivity analysis was performed by eliminating results of two studies with the lowest risk assessment scores.

The sensitivity analysis by removing two studies (Swain et al. and Yarahmadi et al.) (30, 34) with the lowest risk assessment scores, does not alter the original results (mean = 20.53, 95% CI: 18.90, 22.15). The result of the sensitivity analysis is depicted in **Figure 5**.

**Figure 6:**
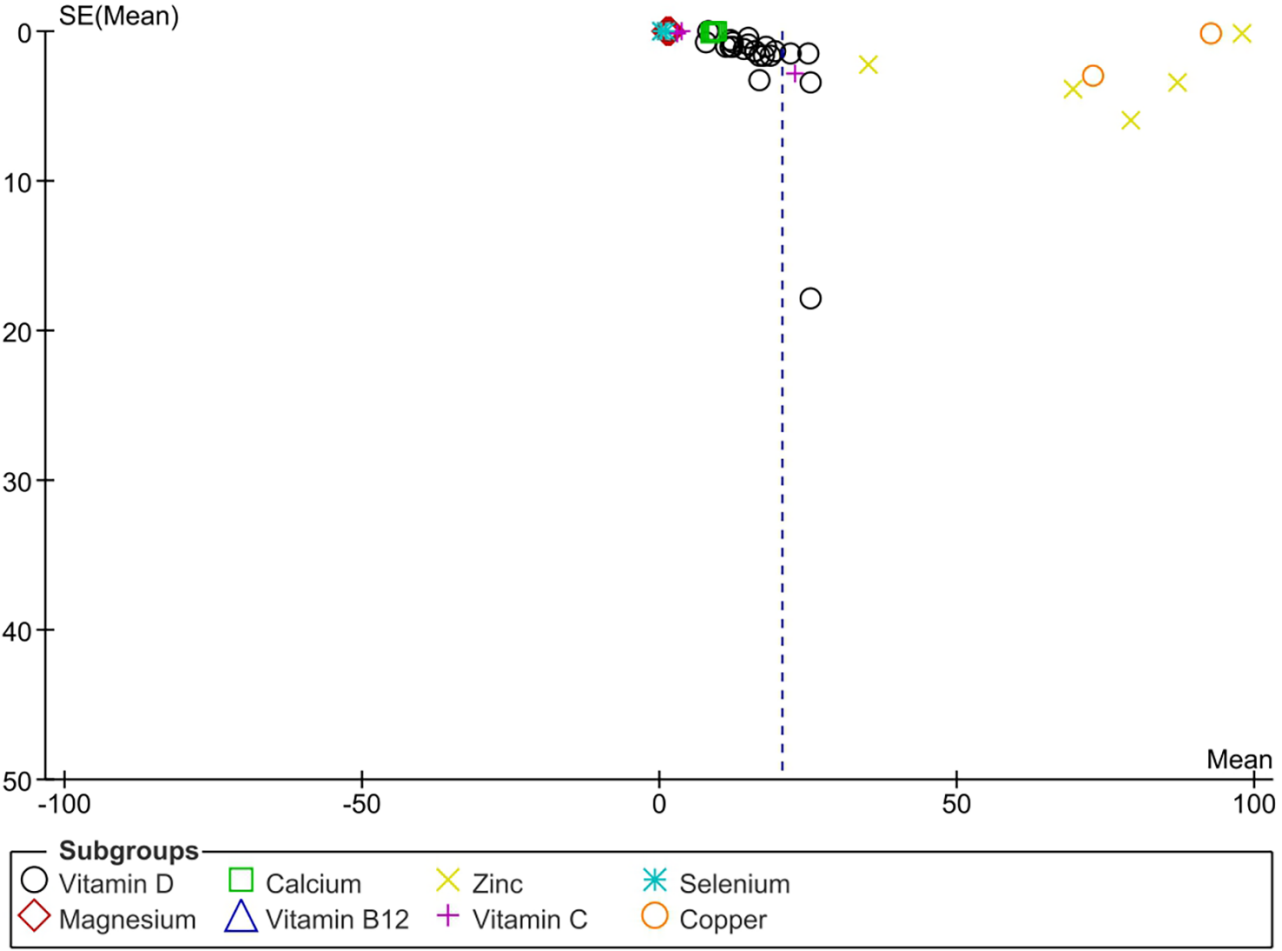
Funnel plot asymmetry test to assess publication bias.

The apparent asymmetry in the funnel plot (**Figure 6**) suggests possible publication bias.

## 4 Discussion

Identifying and managing chronic wounds is a critical healthcare objective. DFU generally starts with minor injuries that go unnoticed because of diabetic neuropathy (altered sensitivity and nerve damage). Convergence of immunological, vascular, nutritional, glycaemic, and infectious conditions influences wound healing. The present meta-analysis has revealed significantly lower circulating levels of vitamin D, vitamin C, magnesium, and selenium among patients with DFU than in control groups. However, other micronutrients did not differ significantly between DFU patients and controls.

Nutritional deficiencies impede normal stages of wound healing, a complex four-step process involving hemostasis, inflammation, proliferation, and tissue remodelling (51). Chronic wounds generally get stalled at the inflammatory phase stage due to the continuous recruitment of neutrophils to the healing site, producing various alterations at systemic and molecular levels. Malnutrition also prolongs the inflammatory phase by decreasing fibroblast proliferation, and collagen formation, in addition to altering its tensile strength and angiogenesis. Malnutrition can increase the risk for infection by reducing T-cell function, phagocytic activity, complement, and antibody levels (52). Nutrients can aid wound healing by minimizing free radicals (neutrophils can release reactive oxygen species) and oxidative stress parameters by balancing the oxidant-antioxidant defenses (53). The higher proportion of nutrient insufficiencies in DFU could disturb glycaemic control, which in turn delays wound healing (49).

Vitamin D is well known for its pleiotropy. Vitamin D deficiency (VDD) is associated with impaired beta-cell function, insulin resistance (54), and micro and macro-vascular complications of DM progression. A recent systematic review and meta-analysis of 1115 patients reported that severe VDD increased DFU risk by 3.2 times (55). Interestingly, Darlington et al. observed similar vitamin D levels between DM and DFU patients but with poor DFU outcomes (25). Pena et al. identified VDD to be dominantly prevalent (55.7%) among DFU patients (6). Dai et al. proposed vitamin D levels below 13.68 ng/ml as the threshold for DFU risk (37).

Vitamin D positively improves immunological, neurological, and vascular conditions associated with DFU. Vitamin D is also an immunomodulator that facilitates T and B cell activation by macrophages. Gupta B and Singh SK showed that macrophages treated with vitamin D3, *in vitro*, enhanced phagocytosis in DFU setting (26). Vitamin D inhibits T-helper cells-1 (Th1) that promote cell-mediated inflammatory response while stimulating Th2 cells that aid wound healing (56). Tiwari et al. suggest 10ng/ml of 25-hydroxy vitamin D [25 (OH)D] as the threshold for immunological alterations in DM. Reports suggest that VDD is associated with an increased release of inflammatory cytokines (TNF-α, IL-1β, IL-6) in DFU patients (48). Vitamin D induces the transcription of cathelicidin and defensins that aid in phagocytosis, thereby enhancing the antimicrobial innate immune system (57).

Asian DM patients with VDD are at 1.22 times greater risk for developing peripheral neuropathy than those with normal vitamin D levels (58). Basit et al. showed that 600,000 IU of vitamin D, over 20 weeks, offered significant pain relief in painful diabetic neuropathy (59). VDD may also be associated with increased sensitivity to pain (60). Swain et al. reported that nearly 52% of DFU patients with vascular calcification (VC) had severe VDD (30). Their subgroup analyses showed that the risk for VC was 2.4 times higher in patients with vitamin D levels < 10 ng/ml. Sugden et al. demonstrated that a single high dose of vitamin D supplementation can improve the flow-mediated vasodilation of the brachial artery by 2.3% (61).

Most studies have focused on the significant role of vitamin D in DFU compared to other nutrients. We need more clinical and molecular studies to explain the results. We identified four clinical trials that estimated 25 (OH) D levels and studied the effects of vitamin D supplementation on DFU outcomes. Kamble et al. and Razzaghi et al. investigated the effect of 60,000 IU and 50,000 IU of vitamin D, respectively, for 12 weeks, in DFU healing (18, 21) and reported that supplements improved wound healing and biochemical parameters. Halschou-Jensen et al. showed that two daily doses (170 µg and 20 µg) of vitamin D supplements in chronic DFU (17) delivered a median ulcer reduction of 100% (high dose) and 57% (low dose). Mozaffari-Khosravi et al. demonstrated that a single dose of 300,000 IU of vitamin D improved DFU outcomes compared to 150,000 IU (22).

Magnesium is an essential element with a pivotal role in human physiology, especially as a cofactor for enzymatic and metabolic pathways (62). Magnesium, essential for collagen formation and tissue development, is altered in DM (63). Hypomagnesemia in DM could result from enhanced renal excretion associated with insulin resistance, glycosuria, and hyperglycemia. Diabetic autonomic neuropathy alters intestinal absorption (27) and reduces dietary intake of magnesium. Improving insulin metabolism can potentially delay vascular complications in DFU. Magnesium plays a role in the formation of malonyl-COA and inhibits voltage-dependent calcium channels that facilitate insulin secretion (20). Hypomagnesemia has been associated with abnormal platelet activity and can induce a proinflammatory response that activates systemic inflammation (64). Hypomagnesemia has also been linked with neuronal damage and diabetic peripheral neuropathy in DM patients (65, 66). Further magnesium supplementation was found to promote peripheral nerve regeneration (67).

Yadav et al. observed an inverse relationship between DM duration and serum magnesium, copper, and zinc levels (49). Rodrigues-Moran et al. provided the first evidence for hypomagnesemia as a risk factor for DFU (OR: 2.9, 95% CI: 1.7-6.8; *P* = 0.01) (46). Interestingly, Moon et al. have reported that hypermagnesemia is a risk factor for amputation in hospitalized DFU patients (OR:2.480; *P*= 0.043), which could be attributed to the association between renal disorder and hypermagnesemia (68).

Two studies have investigated the role of magnesium supplementation in DFU patients. Razzaghi et al. found that 250 mg of magnesium for 12 weeks improved the ulcer area, glycaemic parameters, and other antioxidant and anti-inflammatory parameters (20). Afzali et al. showed that 250mg magnesium plus 400 IU vitamin E can improve ulcer area, glycaemic parameters, lipid profile, and other antioxidant and anti-inflammatory parameters (16). Coger et al. have suggested magnesium supplements during the late-inflammatory and mid-proliferative phases (69).

A population-based cohort study (25,639 participants; 8-12 years) demonstrated an inverse association between vitamin C levels and incidence of DM (70). Vitamin C is a strong antioxidant, a vital co-factor in several enzymatic reactions, and promotes anti-inflammatory and pro-resolution effects in macrophages, together alleviating pro-inflammatory responses (71). Vitamin C deficiency in DM has been established, and its impact on serum malondialdehyde suggests increased oxidative stress, aggravating micro- and macro-vascular complications in DM (72).

A meta-analysis of RCTs shows that vitamin C supplements significantly improved endothelial function in DM. Vitamin C is a direct antioxidant that scavenges reactive oxygen species and enhances the bioavailability of nitric oxide (NO) (73). In 2021, an RCT (n= 16) of vitamin C supplements showed benefits on foot ulcers (74). Inadequate vitamin C supplements can cause stagnation in the proliferative and maturation phases of wound healing, thereby prolonging wound healing time (71). Vitamin C facilitates the synthesis and cross-linking of collagen, enhancing vascular integrity and capillary bed strength (75). Pena et al. identified 73% of DFU patients with suboptimal levels of vitamin C (6). An RCT by Yarahmadi et al. showed that a combination of platelet-rich plasma, fibrin glue dressing, and vitamins E and C improved wound healing of DFU by alleviating oxidative stress (76).

Dixit et al. reported a significant difference between selenium levels in patients with chronic non-healing wounds and HC (77). An *in vivo* study on diabetic mice demonstrated an antioxidant role for selenium (restoring normal antioxidant status), and as an insulin mimetic in normalizing glucose levels. Selenium can also downregulate connexin expression, which promotes anti-inflammatory and anti-apoptotic signals, in addition to enhancing angiogenesis (78). Macrophages treated with selenium promote peroxisome proliferator-activated receptor (PPAR)-γ- dependent switch from M1 to M2 phenotype in the presence of IL-4 (79), suggesting selenium’s wound healing potential.

Currently, available evidence suggests that immune-endocrine effects and antioxidant properties of selenium benefit infections in DM (80). Although we did not identify any interventional studies on the effect of selenium in DFU, selenium levels were markedly different in DFU patients *vis-a-vis* HC and DM (35, 41, 42).

The strength of the current study: This is the first systematic review with meta-analysis comparing micronutrient status in DFU between HC and DM. The limitations are First: relatively small sample size in some studies. Second: most study designs were retrospective or cross-sectional, limiting the possibility of establishing a causal relationship between the micronutrients and DFU. Third: marked publication bias was observed. Fourth: cannot rule out the possibility of ecology and environment as confounders. Nevertheless, the existing challenge is to articulate the effect of these supplementations in the patient population as the number of well-designed RCT’s are few.

We have observed a significant association between DFU and vitamin D, vitamin C, magnesium, copper, and selenium levels. Although other micronutrients also influence multiple phases of wound healing, we did not observe a significant association. Nevertheless, we recommend assessing micronutrient levels in DFU patients and investigating their pathological correlation. Future investigations should address the effect of specific micronutrients in DFU management, molecular mechanisms of action of micronutrients, as well as nutrigenomic studies that reveal gene-nutrient interaction and its possible effects on DFU healing. Individual genetic variants could respond differently to micronutrients, and thus directly or indirectly influence the prevention and management of DFU. Nutrigenomic approaches would deliver a holistic and personalized approach to the management of DFU.

## Data availability statement

The original contributions presented in the study are included in the article/supplementary material. Further inquiries can be directed to the corresponding author.

## Author contributions

SK and SM formulated the research question and designed the study. SK, TB, RB, and SM were involved in carrying out the study, analyzing the data, and interpreting the findings. SK and SM wrote the manuscript. MU, MM, KS, GR, MR, and AK, critically evaluated the manuscript. All authors contributed to the article and approved the submitted version.
